# Intimate Partner Violence and Mental Health: Deepening Our Understanding of Associations, Pathways, and Prevention in Low- and Middle-Income Countries

**DOI:** 10.3390/ijerph20021505

**Published:** 2023-01-13

**Authors:** Lindsay Stark, Ilana Seff, Massy Mutumba, Emma Fulu

**Affiliations:** 1 Brown School, Washington University in St. Louis, St. Louis, MO 63130, USA; 2 Health Behavior and Biological Sciences, University of Michigan School of Nursing, Ann Arbor, MI 48109, USA; 3 The Equality Institute, Melbourne, VIC 3070, Australia

Mental health disorders and related symptoms are among the top leading causes of disability adjusted life years (DALYs) among youth (10–24 years) and adults (25–49 years) [1]. Depressive disorders and self-harm in particular pose a considerable public health burden. In 2019, depressive disorders were the fourth leading cause of DALYs among youth, and the sixth leading cause of death among adults [2]. In addition, self-harm was the third leading cause of DALYs in youth and the eleventh leading cause of DALYs among adults [2]. While mental health adversity is impacted by an ecology of multi-level risk factors, including family history, socio-economic status, and stigma, research and practice have highlighted the unique relationship between interpersonal violence and mental health [3]. Research has analyzed the complex interplay between gender-based violence and mental health to better understand how and why women and girls have a greater burden of internalized mental health disorders (i.e., depression, anxiety, somatic symptoms) compared to men and boys, and how and why men and boys have a greater burden of externalizing behaviors (i.e., substance use disorders, aggression, harmful alcohol use) compared to women and girls [4,5,6,7,8,9].

Such research efforts respond to relevant targets from the 2015–2030 Sustainable Development Goals (SDGs): to eliminate all forms of violence against women and girls (SDG target 5.2.1), to reduce premature mortality from non-communicable diseases due to suicide (SDG target 3.4.2), and to strengthen the prevention and treatment of substance abuse, including harmful alcohol use (SDG target 3.5) [10,11]. Despite research, policy, and practice efforts, forms of gender inequality, such as violence against women and girls, and morbidity and mortality due to mental health disorders, suicide, and substance abuse continue to pose public health and economic challenges, particularly within the context of low- and middle-income countries (LMICs) [10]. In this Special Issue, “Intimate Partner Violence and Mental Health in Low- and Middle-Income Countries”, we present a collection of research articles that elucidate the mechanistic pathways between witnessing, experiencing, and perpetrating intimate partner violence (IPV), and mental health, as well as identifying strategies for successfully mitigating the negative mental health consequences of IPV in LMICs.

Our Special Issue engages specifically with IPV, given this is the most common form of gender-based violence and one that disproportionately affects women and girls in youth and adulthood [12,13]. IPV is defined as harmful acts of abuse or aggression that occur between partners from current or previous romantic relationships [14]. Such violence may include physical, sexual and psychological forms of harmful behavior, including emotional and economic abuse. An estimated 27% of women (15–49 years) will experience physical or sexual IPV in their lifetime [12]. Further, first exposure to IPV begins early: an estimated one in four young women aged 15 to 24 years who have been in a romantic relationship will have already experienced IPV by their mid-twenties [13]. The burden and health consequences of IPV are magnified in settings of instability due to weak economic systems, unemployment, conflict, and eroded social safety nets, as is the case in many LMIC countries [13]. Approximately 37% of women living in the most socio-economically deprived countries will experience physical or sexual IPV in their lifetime [13].

IPV is related to myriad health consequences for women, children, families, and communities [15,16,17,18]. Women and girls who have experienced IPV face a greater risk of sexual and reproductive health adversities, such as sexual risk-taking behaviors, risk of lifetime sexually transmitted infections, unwanted pregnancy or induced abortion, and sexual dysfunction [17], as well as physical health consequences, including acute physical injuries (lacerations, bruising, broken bones) and chronic pain [15,16]. Children who witness IPV between their parents or caregivers are also at risk of intergenerational health disparities including health-compromising behaviors, including alcohol use, cigarette smoking, substance abuse, risky sexual behaviors [19]. These children are also more likely to experience IPV in adulthood, either as perpetrators or as victims [20,21]. Societally, it has been estimated that IPV places a tremendous burden on health and labor costs [22,23].

The mental health consequences of IPV are also significant. A meta-analysis of 17 articles, mainly from high-income countries, reported that women’s IPV exposure was associated with an astounding 97% increase in odds of depressive symptoms [5]. A growing body of literature also points to the mental health impacts of IPV victimization and perpetration in LMICs. In Haiti, exposure to lifetime sexual violence among girls yielded an 80% increase in odds of suicidal ideation, and in Kenya and Tanzania, girls exposed to physical intimate partner violence also had elevated odds of suicidal ideation [8]. An analysis of data from informal settlements in South Africa also found emotional and economic IPV to be predictive of depressive symptoms and suicide ideation [24].

Despite some progress in reducing IPV, and significant progress in elevating the importance of mental health in public discourse, more research is needed to understand the complex interplay between IPV and mental health, particularly in LMICs. In this Special Issue, we present 14 research articles that critically explore these relationships. Contributions to this Special Issue draw on data from across the globe, with studies representing a diverse set of LMICs and generating insights on various marginalized populations, including refugees and sex workers, among others. This Special Issue also widens the lens on the ways in which we think about the impacts of IPV on wellbeing. The studies included employ traditional measures of depression, post-traumatic stress disorder, and suicide ideation [25,26,27,28], but also examine broader psychosocial impacts such as alcohol and drug use, capacity to cope, and resilience [18,27,29,30,31].

Findings presented in this Special Issue expand the evidence base on IPV and mental health in several important ways. First, findings highlight opportunities for practitioners and policymakers to prevent the mental health consequences of IPV earlier in the life course by demonstrating how IPV may mediate the relationships between earlier exposures—such as childhood trauma, HIV+ status, economic precarity, and pandemic conditions—and mental illness [27,28,32,33]. Second, the included analyses elucidate how certain attitudes or behaviors might serve to strengthen or otherwise modify the link between IPV and mental health, and how these interactions may vary by context. For example, while a study from Northern Uganda found that gender equitable attitudes mitigated the relationship between IPV victimization and mental illness [29], analyses from Nigeria revealed that survivors of IPV who believed IPV was acceptable in some situations exhibited lower mental distress [26]. Third, this Special Issue offers actionable guidance for practitioners by evaluating promising intervention approaches [30,34], reviewing the evidence on what works to prevent IPV and subsequent psychological distress [35,36], and highlighting determinants for the successful implementation of integrated interventions that address IPV and mental health in tandem [37]. Importantly, all papers in this Special Issue advance the knowledge base on how to understand and address IPV and mental health in LMICs, ultimately strengthening our ability to make progress toward the SDGs.
